# MicroRNA Profiling of Morphologically Heterogeneous Clear Cell Renal Cell Carcinoma

**DOI:** 10.7150/jca.52310

**Published:** 2021-07-06

**Authors:** Alessio Giubellino, Christopher J. Ricketts, Vanessa Moreno, W. Marston Linehan, Maria J. Merino

**Affiliations:** 1 Translational Surgical Pathology, Laboratory of Pathology, National Institutes of health, Bethesda, MD; 2 Urologic Oncology Branch National Cancer Institute, National Institutes of health, Bethesda, MD

**Keywords:** MicroRNA, Clear cell renal cell carcinoma, ccRCC, Intratumoral heterogeneity, Tumor histology, Heterogeneous tumor morphology

## Abstract

Intratumoral heterogeneity (IH) has been recently described as an important contributor to tumor growth through a branched rather than a linear pattern of tumor evolution for renal cell carcinoma. As to whether the miRNA profiling of the different and heterogeneous areas is the same or not, it is not known. This study analyzed the differences and similarities of the miRNA profiles in histologically distinct regions within several RCC tumors. The observed differences may have great implications for the development of predictive biomarkers and the identification of druggable targets with improvement of combinatorial therapeutic approaches for the effective treatment of kidney cancer, as well as for the identification of circulating malignant cells that can be useful to detect tumor recurrences.

## Introduction

Renal cell carcinoma (RCC) represents the most common epithelial kidney tumor and the seventh most common cancer, accounting for about 3% of all adult malignancies 1. In 2014, approximately 270,000 patients worldwide were diagnosed with RCC and 120,000 patients died from the disease 2. RCC is a heterogeneous neoplasm consisting of a number of different types of cancer that have been characterized by histopathologic presentation and are associated with different genetic and molecular backgrounds 3. The study and characterization of renal cell carcinoma is a remarkable story of accomplishments in oncology research, since the discovery of the genetic basis of the disease has greatly advanced the way patients with advance disease are treated 4. The classification of RCC based on a combination of histopathology, genetic analysis and molecular markers has been extremely important in predicting patient outcomes and in the selection of better, more targeted therapeutic options 4-6. Further improvements to the current characterization and classification of RCC tumors should help to refine both prognosis and treatment.

The recent discovery of intratumor heterogeneity within the most common form of RCC, clear cell renal cell carcinoma (ccRCC), has had a major impact on the characterization of ccRCC. The majority of ccRCC tumors demonstrate the same main driving feature of loss of VHL function that is present throughout the tumor 7, 8. Further mutation studies have demonstrated that, although VHL loss remained constant, the acquisition of additional somatic changes can vary between different regions of a ccRCC tumor 7, 8. It is important to recognize that different regions of a tumor may exhibit different additional genetic or molecular alterations that may need different types of therapeutic intervention as well as identifying features that are universal to the tumor and potentially present as a therapeutic target for the entire tumor. Current research has demonstrated that different regions of a ccRCC tumor may contain varying mutational spectrums, but these regions may also demonstrate differing mRNA or miRNA profiles. In addition, ccRCC tumors can present with multiple histopathologic patterns that define morphologically different regions of a tumor that may reflect different mutation or RNA profiles.

Several previous studies have characterized the expression profiles of specific miRNAs in the pathophysiology of renal cell carcinoma 9, 10. Increased expression of miR-210 and miR-21 are associated with ccRCC and correlate with adverse outcome 11. Furthermore, increased expression, due to decreased promoter methylation, of miR-21 may contribute to the glycolytic shift observed in ccRCC and the activation of the PI3K/AKT pathway and this increased expression was correlated with decreased overall survival 12. Growing numbers of studies are demonstrating the importance of miRNAs in the control of physiologic cellular processes and their role in the etiology of human diseases 13, 14. MicroRNA expression signatures are now being used to classify several types of tumor at the molecular level 15-17.

This study investigated the differences between miRNA expression profiles from histopathologically-defined, morphologically different regions within ccRCC tumors. This study demonstrated that it was possible to characterize each different morphologic region of ccRCC tumors based uniquely on their miRNA profiles. These finding further emphasize that the heterogeneity observed within ccRCC tumors is present at the molecular level and may be associated with histologic pattern, which may have important prognostic and predictive value for therapeutic intervention.

## Material and Methods

**Tissue samples and microdissection:** Four cases of ccRCC were selected based on H&E staining analysis demonstrating distinct morphologic tumor heterogeneity. Ten micron-thick unstained slides were obtained from formalin fixed paraffin embedded (FFPE) tumors. Tissue was procured by microdissection from different areas of the slide that represented the different morphological regions. These regions were classified for each tumor as described in the Results section and were designated by a number related to the patient case ID number. A region of normal renal parenchyma was micro-dissected from one of the four cases (Patient 3) where material sufficiently distant from the tumor was available.

**RNA isolation:** Total RNA was extracted using RecoverAll™ Total Nucleic Acid Isolation Kit (Ambion, Austin, TX, USA) according to manufacturer's instructions. The total RNA was treated with DNase (Ambion, Austin, TX, USA) and the RNA concentration was evaluated by spectrophotometry using a NanoDrop ND-1000 (Thermo Scientific, Waltham, MA, USA).

**MicroRNA array profiling and analysis:** MicroRNA expression profile for the total RNAs extracted from the different morphological regions and the normal control were evaluated using nCounter Human v2 miRNA Expression Assay (NanoString Technologies, Seattle, WA, USA ). The raw data was analyzed using nSolver Analysis Software (V.1.1) and tabulated in Excel. Background correction was performed by calculation the average background signal produced by the negative controls and then subtracting this value from the raw signal. All background corrected values that were negative were converted to zero and represented no detectable expression of the miRNA. The background corrected values were normalized across samples using the averaged mRNA expression values for three housekeeping genes, *ACTB*, *RPL19*, and *RPLP0*. The relative expression of these three housekeeping genes within each of the tumor regions was compared to their expression level within the normal kidney parenchyma sample (considered to have a value of 1.00). The average of the relative expression values for these three genes within each tumor region was then used to normalize all the miRNA expression values within each tumor. Once normalized, a relative miRNA expression value was calculated for each miRNA in each tumor region in comparison to the normal kidney parenchyma miRNA expression. MicroRNAs that demonstrated 2-fold or greater up- or down-regulation were considered to be significantly altered relative to normal kidney parenchyma. MicroRNAs that were expressed in the tumor but not in the normal or expressed in the normal but lost in the tumor were designated as “Only in Tum.” or “Lost in Tum.” respectively and were considered significant. Venn diagrams were created using software available from the Whitehead Institute for Biomedical Research (http://jura.wi.mit.edu/bioc/tools/).

**Cluster analysis:** To perform cluster analysis between all the different morphological regions, the average normalized miRNA expression values were calculated across the tumor regions. The relative tumor miRNA expression for each miRNA for each tumor region was calculated in comparison to the average miRNA expression across the tumors. Cluster analysis was performed on this data using the Gene Cluster 3.0 software (http://bonsai.hgc.jp/~mdehoon/software/cluster/software.htm) to evaluate hierarchical Euclidean clustering with average linkage and this was visualized using TreeView (http://jtreeview.sourceforge.net/).

## Results

### Heterogeneous histopathologic morphologies within clear cell renal cell carcinomas

**Figure 1:**
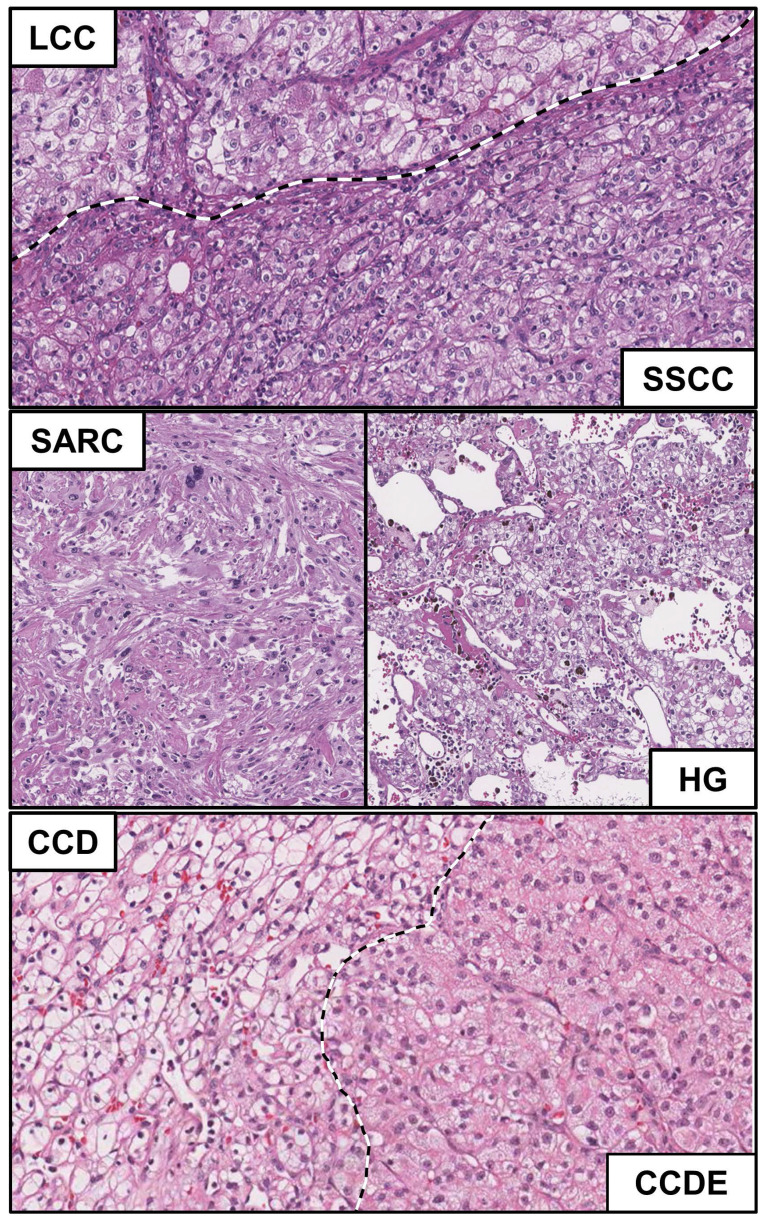
** Histopathologic evaluation of morphological heterogeneity with ccRCC.** Microscopic features of the different morphological pattern of clear cell renal cell carcinoma. Normal=N; Sarcomatoid = SARC; Large clear cell = LCC; Solid small clear cell = SSCC; High grade = HG; Clear cell dense eosinophilic nodule = CCDE; Clear cell diffuse = CCD.

The laboratory of pathology at the National Cancer Institute has a vast collection of clear cell renal cell carcinomas from which four tumors from four individual patients were selected that demonstrated heterogeneous histopathologic morphologies. In each case, at least two separate morphologically different regions of the tumor were identified and materials from these regions could be acquired. Six different histopathologic morphologies were identified consisting of four different types of clear cell-like patterns that were designated as either large, solid small, dense eosinophilic nodule, or diffuse and two further patterns that were designated as sarcomatoid and high grade (Table 1). In comparison to the conventional clear cell histologic presentation that has a classic alveolar pattern and clear cytoplasm, the large clear cell (LCC) morphology has abundant granular cytoplasm (Figure 1- upper panel, upper portion), the solid small clear cell (SSCC) has a more compact architecture made of smaller cells (Figure 1 - upper panel, lower portion), the clear cell with dense eosinophilic nodule (CCDE) has granular eosinophilic cytoplasm (Figure 1 - lower panel, right) and the clear cell diffuse (CCD) has a clear cell cytoplasm but not alveolar growth pattern (less thin walled blood vessels) (Figure 1 - lower panel, left). The sarcomatoid pattern (SARC) is a highly cellular lesion with spindle-like cells and no epithelial component (Figure 1 - middle panel, left), while the high grade pattern (HG) has nuclear pleomorphism, often with multi-lobed nuclei and heavy chromatin clumps (Figure 1 - middle panel, right).

In this cohort, the three tumors from patients 1, 2, and 4 demonstrated two different morphologies and the tumor from patient 3 demonstrated three separate morphologies with the LCC morphology representing the most commonly observed morphology (Table 1). Patients had an average age of 61.5 years at diagnosis and consisted of three male patients and one female patient (Table 1). Notably, the tumors were relatively large with an average greatest dimension of 6.95 cm and demonstrated a tendency towards having a higher nuclear grade (Table 1).

### Characterization of miRNA expression profiles in different histopathologic morphologies

**Figure 2:**
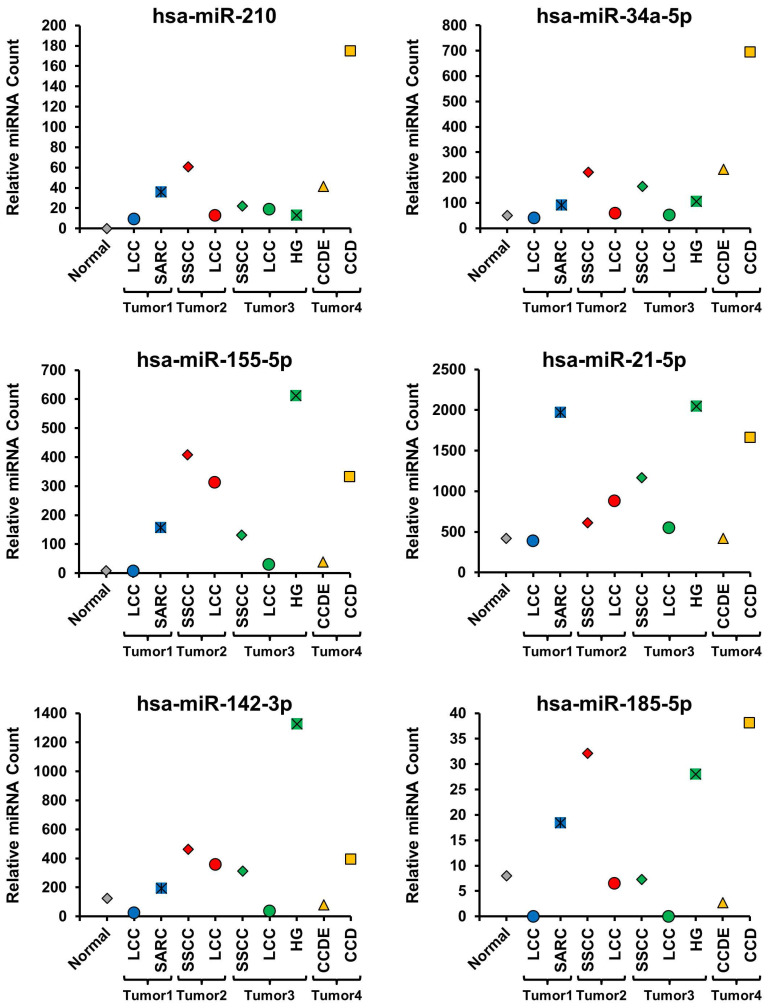
** Evaluation of miRNAs known to be up-regulated in ccRCC.** The expression levels of 6 miRNAs known to be up-regulated in ccRCC were compared across all morphologic regions of the tumors and with the normal tissue. Tumors were coded blue for tumor 1, red for tumor 2, green for tumor 3, and yellow for tumor 4 and the normal was colored gray.

**Figure 3:**
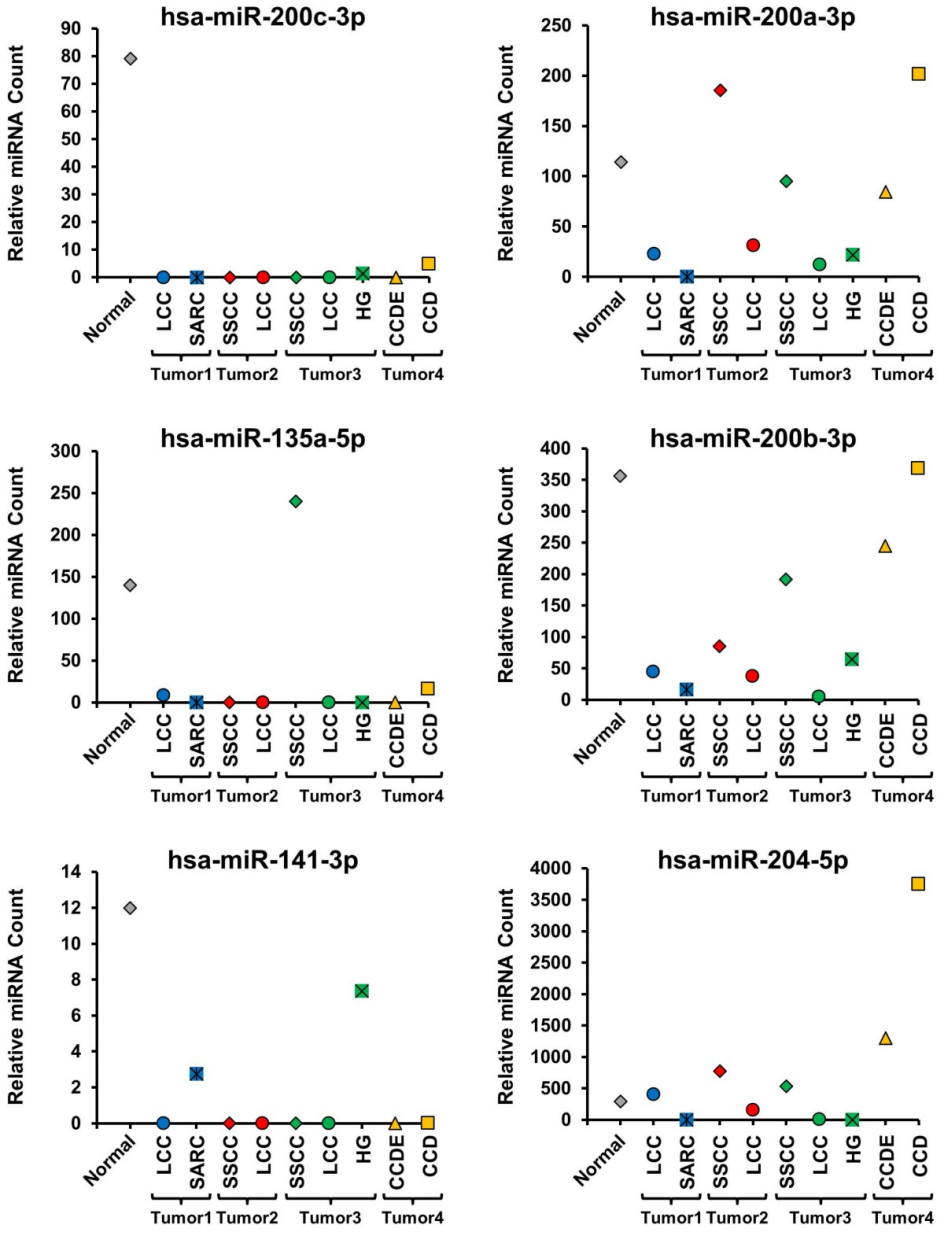
** Evaluation of miRNAs known to be down-regulated in ccRCC.** The expression levels of 6 miRNAs known to be down-regulated in ccRCC were compared across all morphologic regions of the tumors and with the normal tissue. Tumors were coded blue for tumor 1, red for tumor 2, green for tumor 3, and yellow for tumor 4 and the normal was colored gray.

All the different histopathologic morphological patterns were separately isolated by macro-dissection and evaluated for miRNA expression. Initially, 6 miRNAs known to be up-regulated in ccRCC (hsa-miR-210, hsa-miR-34a-5p, hsa-miR-155-5p, hsa-miR-21-5p, hsa-miR-142-3p, and hsa-miR-185-5p) and 6 miRNAs known to be down-regulated in ccRCC (hsa-miR-200c-3p, hsa-miR-200a-3p, hsa-miR-135-5p, hsa-miR-200b-3p, hsa-miR-141-3p, and hsa-miR-204-5p) were selected and compared between the different histopathologic morphologic regions and the normal renal parenchyma (Figure 2 and 3) 18. This demonstrated distinct differences between the different morphologic regions within the same tumor as well as some patterns that correlated with specific morphologies (Figure 2 and 3, Supplementary Table 1). Hsa-miR-210 expression is known to be up-regulated by the activation of the HIF1α mediated hypoxia signaling pathway that is a hallmark of ccRCC and was undetectable in the normal parenchyma, but expressed to varying degrees in all morphologic regions of all the tumors (Figure 2) 19. Within tumors, different morphologic regions demonstrated different expression profiles for the majority of the selected up-regulated miRNAs. In tumor 1, the SARC region consistently demonstrated increased expression of the up-regulated miRNAs in comparison to the LCC region (Figure 2). In tumor 2, the SSCC region demonstrated slightly increased expression for the majority of the up-regulated miRNAs compared to the LCC region, except for hsa-miR-21-5p that was more highly expressed in the LCC region (Figure 2). In tumor 3, all three regions showed similar expression of hsa-miR-210, while the SSCC region showed the highest expression of hsa-miR34a-5p and the HG region demonstrated significantly higher expression of the remaining four up-regulated miRNAs in comparison to the other two regions (Figure 2). In tumor 4, the CCD region consistently demonstrated greatly increased expression of the up-regulated miRNAs in comparison to the CCDE region, similar to the pattern observed in tumor 1 (Figure 2). Two of the down-regulated miRNAs (hsa-miR-200c-3p, hsa-miR-135a-5p, and hsa-miR-141-3p) demonstrated almost universal loss of expression in all region of all tumors with only the SSCC region of tumor 3 expressing hsa-miR-135a-5p (Figure 3). Has-miR-141-3p loss expression in most regions but only had reduced expression in the SARC region of tumor 1 and the HG region of tumor 3 (Figure 3). The remaining three selected down-regulated miRNAs demonstrated the same patterns of heterogeneity within each tumor. In tumor 1, the SARC region consistently demonstrated increased down-regulation of hsa-miR-200a-3p, hsa-miR-200b-3p, and hsa-miR-204-5p in comparison to the LCC region (Figure 3). While in tumors 2 and 3, the LCC regions demonstrated lower expression levels of these miRNAs than the SSCC regions and for hsa-miR-200a-3p and hsa-miR-204-5p the SSCC regions demonstrated little or no down-regulation (Figure 3). In tumor 4, the CCD region consistently demonstrated increased or normal expression of the down-regulated miRNAs in comparison to the normal renal parenchyma, while the CCDE region showed some down-regulation of these miRNAs (Figure 3).

**Figure 4:**
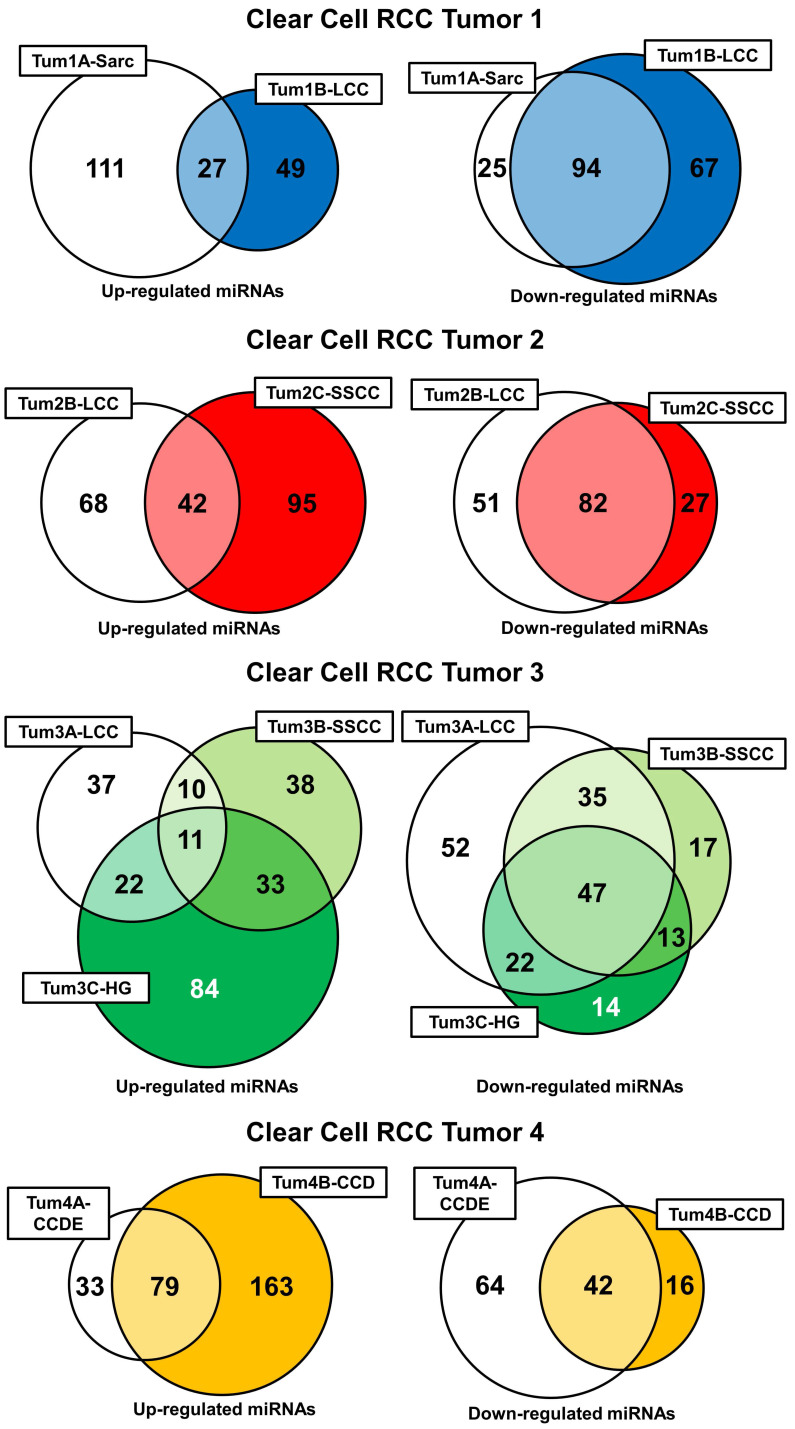
** Intratumoral comparison of miRNA expression profiles.** Venn diagrams demonstrate the number of shared and unique up- or down-regulations of miRNAs within each region of each tumor. Each tumor was evaluated individually and colored-coded blue for tumor 1, red for tumor 2, green for tumor 3, and yellow for tumor 4.

Further evaluation of the miRNA expression profiles was performed by comparing the expression levels of all the miRNAs between the different histopathologic morphologic regions and the normal renal parenchyma with a 2-fold up- or down-regulation considered significant (Supplementary Table 1). This produced a list of up- and down regulated genes for each different morphologic region that could be compared within each tumor (Supplementary Table 2). In tumors 1 and 2 the different morphologic regions shared a significant number of down-regulated miRNAs (50.5% and 51.3% respectively), but each had a more divergent pattern of miRNA up-regulation (Figure 4). Tumor 3 demonstrated a similar pattern with all three different morphologic regions sharing 23.5% of the down-regulated miRNAs and only 4.7% of the up-regulated miRNAs and with at least 2 different regions sharing 58.5% of the down-regulated miRNAs (Figure 4). Interestingly, the SARC region of tumor 1 and the HG region of tumor 3 both demonstrated a large number of specific miRNA up-regulations, but few specific miRNA down-regulations. Tumor 4 demonstrated similar levels of shared up- and down-regulated miRNAs with the CCD region demonstrating a larger number of miRNA up-regulations and the CCDE region demonstrating a larger number of miRNA up-regulations (Figure 4). Comparison of the LCC regions in tumors 1, 2, and 3 and the SSCC regions in tumors 2 and 3 demonstrated very few miRNA alterations were shared by the histologically similar regions (Supplementary Table 3). The large clear cell (LCC) regions had one miRNA commonly up-regulated, hsa-miR-572, and two miRNA commonly down-regulated, hsa-miR-24-3p and hsa-miR-1180. While the solid small clear cell (SSCC) regions pattern only had one miRNA commonly up-regulated, hsa-miR-646.

Each morphologic region demonstrated unique miRNA dys-regulations that were not observed in any other morphologic region of any other tumor (Supplementary Table 4). The majority of these differences involved the up-regulation of miRNAs and several morphologic regions demonstrated no unique miRNA down-regulations. Notably, hsa-miR-95 that is a proliferation-promoting miRNA was uniquely up-regulating in the SARC region of tumor 1 and hsa-miR-569 that is involved in survival of proliferation of tumor cells by downregulating p53 expression was uniquely up-regulated in the HG region of tumor 3 (Supplementary Table 4).

**Figure 5:**
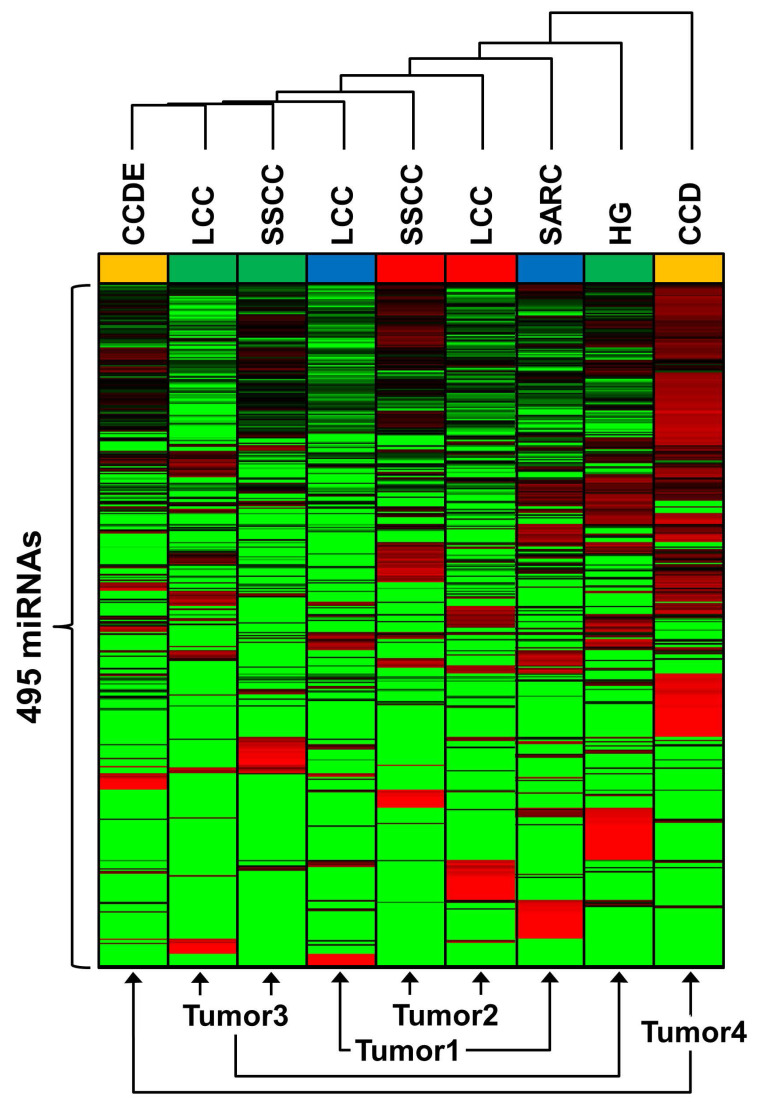
** Comparative expression of miRNA between tumor regions.** Unsupervised Euclidean clustering was performed on 495 miRNAs that all demonstrated expression in a minimum of one region within any tumor. Red denotes increased expression in comparison to the other tumor regions and green denotes decreased expression in comparison to the other tumor regions. Each tumor was colored-coded blue for tumor 1, red for tumor 2, green for tumor 3, and yellow for tumor 4.

To demonstrate the degree variation between both the tumors and the different morphologic regions unsupervised hierarchical Euclidean clustering of 495 miRNAs expressed in a minimum of one tumor was performed (Figure 5, Supplementary Table 5). The relative expression of each miRNA for each morphologic region was calculated in comparison to the average expression value across all regions in all tumors. Distinct miRNA profiles were observed for all the morphologic regions for every tumor. Although the LCC and SSCC regions of tumors 2 and 3 did cluster next to each other, the SARC region of tumor 1 and the HG region of tumor 3 clustered away from the other morphologic regions of their respective tumors. The CCD and CCDE regions of tumor 4 diverged greatly from each other and represent the most significantly different miRNA profiles seen within one tumor (Figure 5).

## Discussion

Intratumoral heterogeneity (IH) is an important feature of clear cell renal cell carcinoma (ccRCC) and may play a very important role in the characterization and therapeutic treatment of ccRCC. Several studies have demonstrated intratumor heterogeneity within the somatic mutational profile of ccRCCs, (Supplemental Figure 1) while no studies have been performed to demonstrate intratumor heterogeneity within the miRNA expression profiles of ccRCC 7, 8. Additionally, the previous studies of mutational heterogeneity within a ccRCC tumor selected the different regions on a non-specific basis by picking region that were simply spatially distinct 7, 8. This is the first study to investigate the miRNA profiles in heterogeneous ccRCC and to relate any heterogeneity to different regions within a ccRCC tumor defined by distinct histopathologic morphology patterns.

This study demonstrated intratumor heterogeneity of the miRNA expression profiles within the different morphologic regions of all four evaluated tumors. Although different morphologic regions within a tumor did share some common miRNA dysregulations in comparison to normal kidney, each region demonstrated unique patterns of expression for some miRNAs. In general, down-regulation of miRNAs were more commonly shared between different morphologic regions, while up-regulation of miRNAs demonstrated a greater degree of variation between regions. Furthermore, unique alterations across all morphologic regions of all the studied tumors were observed in each sample studied. Comparison of tumors with regions of similar histopathologic morphology did not identify much overlap in miRNA dysregulation, although the down-regulation of hsa-miR-646 that was shared by solid small clear cell regions has been previously associated with metastasis in ccRCCs 20. These observations are obviously limited by the number of tumors and regions that were evaluated. Further analysis with increased numbers of samples may demonstrate that few dysregulations are truly unique to a tumor and that a greater degree of miRNA dysregulation is shared by samples with similar histopathologic morphology. One caveat to this study is that multiple samples were not taken from each of the different morphologic regions and so it could not be evaluated if samples from the same morphologic region would demonstrate a high degree of homogeneity.

Evaluation of several miRNAs known to be associated with ccRCC, such as Hsa-miR-210, also demonstrated intratumor heterogeneity 11, 18. Interestingly, miRNAs like Hsa-miR-210 are regulated by the hypoxia response pathway, that is commonly up-regulated in ccRCCs due to loss of the VHL protein complex, and are expected to be over-expressed 9, 19, 21. Hsa-miR-210 expression was increased in all regions but the observed variation in expression within tumors suggests that different regions may be experiencing different levels of hypoxia response or that factors other than the hypoxia response pathway are regulating this miRNA. Notably in all three regions of tumor 3 the Hsa-miR-210 expression levels were similar, even in the high-grade region, demonstrating that similar conditions can occur within all regions of a tumor. This study confirmed the up- and down regulation of several miRNAs previously associated with ccRCC 18. Notably, hsa-miR-21-5p demonstrated significantly increased expression in the sarcomatoid region of tumor 1 and the high grade region of tumor 3 and up-regulation of this miRNA has been associated with poor cancer-specific survival 11, 22. This finding may also have implications in the response to therapy; indeed, the presence and percentage of sarcomatoid dedifferentiation has been linked to response to selective targeted therapy 23, 24. Nearly all regions of all tumors demonstrated down-regulation of hsa-miR-200c-3p, a suppressor of the CDK2 cell-cycle progression regulator, hsa-miR-135a-5p, a suppressor of the MYC oncogene, and hsa-miR-141-3p, a regulator of proliferation and metastasis in ccRCC 25-27. Down-regulation of hsa-miR-200c-3p and hsa-miR-141-3p have been associated with poor cancer-specific survival and knowledge of their targets could add in designing therapeutic regimes 11.

As observed in the studies on somatic mutation intratumor heterogeneity, these patients presented with relatively large tumors with an average largest dimension of ~7 cm. The large size of these tumors is likely to correlate with a long time period of growth and development before diagnosis and thus provide the opportunity for the development of different morphologically distinct regions and intratumor heterogeneity. Further studies of small tumors may demonstrate lower rates of intratumor heterogeneity, but it is the larger tumors that are more likely to metastasize and require systematic therapeutic intervention.

The recognition that different morphological patterns (IH) inside the same tumor are associated with distinct miRNA signatures may have great implications for the development of predictive biomarkers and provides additional evidence for the importance of multiple sampling in the characterization of tumors. MicroRNA signatures could be useful in supporting the diagnostic algorithm of kidney cancer, by offering an ancillary molecular study which can help to support the morphological diagnosis. Furthermore, specific miRNA alterations could be associated with specific druggable targets leading to improvement of combinatorial therapeutic approaches for a more effective treatment of kidney cancer.

## Supplementary Material

Supplementary tables.

## Figures and Tables

**Table 1:** Clinicopathologic characteristic of the patients and relative samples used for miRNA profiling**.**

Case ID	Age of Diag.	Gender	Histologic Type	Morphologic Patterns	Size (cm)	Nuclear Grade	Laterality	Tumor Focality
Patient 1	64	M	Clear Cell RCC	Sarcomatoid, Large Clear Cell	10x8x5	4	Left	Multifocal
Patient 2	49	M	Clear Cell RCC	Large Clear Cell, Solid Small Clear Cell	6.5x5x5	3	Right	Unifocal
Patient 3	68	M	Clear Cell RCC	Large Clear Cell, Solid Small Clear Cell, High Grade	7.5x6x3	4	Right	Unifocal
Patient 4	65	M	Clear Cell RCC	Clear Cell Dense Eosinophilic, Clear Cell Diffuse	3.8x2.5x1.2	2, 3	Left	Multifocal

## References

[1] Siegel R, Ma J, Zou Z, Jemal A (2014). Cancer statistics, 2014. CA Cancer J Clin.

[2] Sourbier C, Srinivasan R, Linehan WM (2015). Metabolism and oxidative stress response pathways in kidney cancer: a tale of chance and necessity. Am Soc Clin Oncol Educ Book.

[3] Moch H, Cubilla AL, Humphrey PA, Reuter VE, Ulbright TM (2016). The 2016 WHO Classification of Tumours of the Urinary System and Male Genital Organs-Part A: Renal, Penile, and Testicular Tumours. Eur Urol.

[4] Linehan WM, Ricketts CJ (2014). Decade in review-kidney cancer: discoveries, therapies and opportunities. Nat Rev Urol.

[5] Capitanio U, Montorsi F (2016). Renal cancer. Lancet.

[6] Haddad AQ, Margulis V (2015). Tumour and patient factors in renal cell carcinoma-towards personalized therapy. Nat Rev Urol.

[7] Gerlinger M, Rowan AJ, Horswell S, Larkin J, Endesfelder D, Gronroos E (2012). Intratumor heterogeneity and branched evolution revealed by multiregion sequencing. New England Journal of Medicine.

[8] Gerlinger M, Horswell S, Larkin J, Rowan AJ, Salm MP, Varela I (2014). Genomic architecture and evolution of clear cell renal cell carcinomas defined by multiregion sequencing. Nat Genet.

[9] Valera VA, Walter BA, Linehan WM, Merino MJ (2011). Regulatory Effects of microRNA-92 (miR-92) on VHL Gene Expression and the Hypoxic Activation of miR-210 in Clear Cell Renal Cell Carcinoma. J Cancer.

[10] Grange C, Collino F, Tapparo M, Camussi G (2014). Oncogenic micro-RNAs and Renal Cell Carcinoma. Front Oncol.

[11] Tang K, Xu H (2015). Prognostic value of meta-signature miRNAs in renal cell carcinoma: an integrated miRNA expression profiling analysis. Sci Rep.

[12] Cancer Genome Atlas Research N (2013). Comprehensive molecular characterization of clear cell renal cell carcinoma. Nature.

[13] Jonas S, Izaurralde E (2015). Towards a molecular understanding of microRNA-mediated gene silencing. Nat Rev Genet.

[14] Londin E, Loher P, Telonis AG, Quann K, Clark P, Jing Y (2015). Analysis of 13 cell types reveals evidence for the expression of numerous novel primate- and tissue-specific microRNAs. Proc Natl Acad Sci U S A.

[15] Lin S, Gregory RI (2015). MicroRNA biogenesis pathways in cancer. Nat Rev Cancer.

[16] Kasinski AL, Slack FJ (2011). Epigenetics and genetics. MicroRNAs en route to the clinic: progress in validating and targeting microRNAs for cancer therapy. Nat Rev Cancer.

[17] Seven M, Karatas OF, Duz MB, Ozen M (2014). The role of miRNAs in cancer: from pathogenesis to therapeutic implications. Future Oncol.

[18] Juan D, Alexe G, Antes T, Liu H, Madabhushi A, Delisi C (2010). Identification of a microRNA panel for clear-cell kidney cancer. Urology.

[19] Kulshreshtha R, Ferracin M, Wojcik SE, Garzon R, Alder H, gosto-Perez FJ (2007). A microRNA signature of hypoxia. Molecular and Cellular Biology.

[20] Li W, Liu M, Feng Y, Xu YF, Huang YF, Che JP (2014). Downregulated miR-646 in clear cell renal carcinoma correlated with tumour metastasis by targeting the nin one binding protein (NOB1). Br J Cancer.

[21] Rustum YM, Chintala S, Durrani FA, Bhattacharya A (2018). Non-Coding Micro RNAs and Hypoxia-Inducible Factors Are Selenium Targets for Development of a Mechanism-Based Combination Strategy in Clear-Cell Renal Cell Carcinoma-Bench-to-Bedside Therapy. International journal of molecular sciences.

[22] Vergho D, Kneitz S, Rosenwald A, Scherer C, Spahn M, Burger M (2014). Combination of expression levels of miR-21 and miR-126 is associated with cancer-specific survival in clear-cell renal cell carcinoma. BMC Cancer.

[23] Beuselinck B, Lerut E, Wolter P, Dumez H, Berkers J, Van Poppel H (2014). Sarcomatoid dedifferentiation in metastatic clear cell renal cell carcinoma and outcome on treatment with anti-vascular endothelial growth factor receptor tyrosine kinase inhibitors: a retrospective analysis. Clinical genitourinary cancer.

[24] Golshayan AR, George S, Heng DY, Elson P, Wood LS, Mekhail TM (2009). Metastatic sarcomatoid renal cell carcinoma treated with vascular endothelial growth factor-targeted therapy. Journal of clinical oncology: official journal of the American Society of Clinical Oncology.

[25] Wang X, Chen X, Han W, Ruan A, Chen L, Wang R (2015). miR-200c Targets CDK2 and Suppresses Tumorigenesis in Renal Cell Carcinoma. Mol Cancer Res.

[26] Yamada Y, Hidaka H, Seki N, Yoshino H, Yamasaki T, Itesako T (2013). Tumor-suppressive microRNA-135a inhibits cancer cell proliferation by targeting the c-MYC oncogene in renal cell carcinoma. Cancer Sci.

[27] Chen X, Wang X, Ruan A, Han W, Zhao Y, Lu X (2014). miR-141 is a key regulator of renal cell carcinoma proliferation and metastasis by controlling EphA2 expression. Clin Cancer Res.

